# Anesthetic Considerations for Cesarean Section in a Woman With Systemic Mastocytosis

**DOI:** 10.7759/cureus.92864

**Published:** 2025-09-21

**Authors:** William Phipps, Lydia Richardson, Erica Jolly

**Affiliations:** 1 Anesthetics, Southampton General Hospital NHS Foundation Trust, Southampton, GBR

**Keywords:** allergy and anaphylaxis, cesarean section, high-risk pregnancy, obstetric anesthesia, obstetric regional anesthesia, peri-operative allergy, peri-operative anesthesia service, systemic mastocytosis

## Abstract

Mastocystosis is a multisystem disorder characterized by mast cell deposition throughout internal organs. It can present in a similar fashion to anaphylaxis, with widespread mast cell degranulation triggered by a range of drugs, sleep deprivation, foods, and physical or emotional stress, such as pain or infection. We present a case of a woman in her 20s with her second pregnancy with suspected mastocytosis who required an elective cesarean section, and consider the implications of anesthetic management. A “recipe” with rationale has been devised to aid others who may come across a similar case in both the elective and emergency settings.

## Introduction

Systemic mastocytosis (SM) is a rare condition, with an estimated prevalence of 1-5 cases per 10,000 [1]. Characterized by the deposition of mast cells throughout extra-cutaneous organs, it can be subdivided into four categories [2]. Diagnosis is based on the WHO major and minor criteria, according to histological and biochemical findings [3]. There is a requirement for one major and one minor criterion or at least three minor criteria to make a diagnosis of SM.

The clinical manifestations of SM are largely secondary to widespread mast cell degranulation and histamine release. They can cause life-threatening problems with the airway, breathing, and circulation, with throat swelling, bronchospasm, vasodilatation, and hypotension [4]. Non-histamine-related issues include bone pain and muscle pain, osteopenia, and osteoporosis. Triggers are varied and can include changes in temperature, emotional and physical stress, as well as a multitude of drugs. For these reasons, both labor and the peri-operative period can be high risk with potential for multifactorial acute, life-threatening reactions. Anesthetic management, therefore, needs to focus on mitigating risk and maintaining vigilance for manifestations of disease. A clear plan for what should and should not be considered is imperative for reducing that risk.

## Case presentation

The patient, in her 20s, first presented to midwifery services at nine weeks pregnant. She was gravida two with a parity of one. Her first child was delivered by emergency cesarean section under spinal anesthesia with subsequent postpartum hemorrhage likely due to placental abruption, and postnatal hyperthyroidism. She was referred to the maternal medicine service primarily due to a diagnosis of Ehlers-Danlos syndrome, for which her main symptom was tachycardia, with a normal 24-hour ECG and normal echocardiogram. Her other past medical history included L5-S1 spinal fusion and multiple ankle and foot surgeries. During pregnancy, she was on atenolol 25 mg once a day (switched from ivabradine during pregnancy), amitriptyline 30 mg once at night, folic acid 5 mg once daily, and co-codamol 30 mg/500 mg as required.

During her orthopedic surgeries, she experienced several incidences of adverse reactions to drugs, with it often being unclear which drug was the culprit. Her first reaction was at the age of 11 years, and she had an anaphylactic reaction during her spinal surgery. Because of this, the patient carried an adrenaline autoinjector and had documented allergies and intolerances to multiple drugs, including antibiotics and chlorhexidine, which had been confirmed on allergy testing. Despite testing and avoidance of triggers, during a subsequent elective ankle operation, she developed a suspected reaction to gentamicin, which had previously been well tolerated. Again, she was referred to the allergy service five months before becoming pregnant with her second child, as she required further ankle surgery. Of note, she intermittently suffered from hot flushes and gastrointestinal upset. Blood tests were taken for IgE to penicillin, cephalosporin, and chlorhexidine, as well as a baseline tryptase. These were all normal, and she was referred to the allergy multidisciplinary team (MDT). Here, a decision was made to test for c-KIT mutation. This returned an equivocal result, and based on this, as well as the clinical presentation, it was suggested that the patient be treated as having confirmed systemic mastocytosis (SM) until a bone marrow biopsy could be obtained postnatally.

The patient’s case was subsequently reviewed in peri-operative allergy MDT and maternal medicine MDT with a plan to deliver via elective cesarean section at 38 weeks. She presented overnight at 35 weeks with threatened pre-term labor. The peri-operative allergy MDT plan was consulted, and a theatre was set aside with all chlorhexidine-containing products removed should there be a need for emergency intervention. She was observed for 24 hours and discharged home once contractions had settled spontaneously. At this point, she was started on fexofenadine 180 mg twice a day with a plan to continue this until her elective cesarean section at 38 weeks.

**Figure 1:**
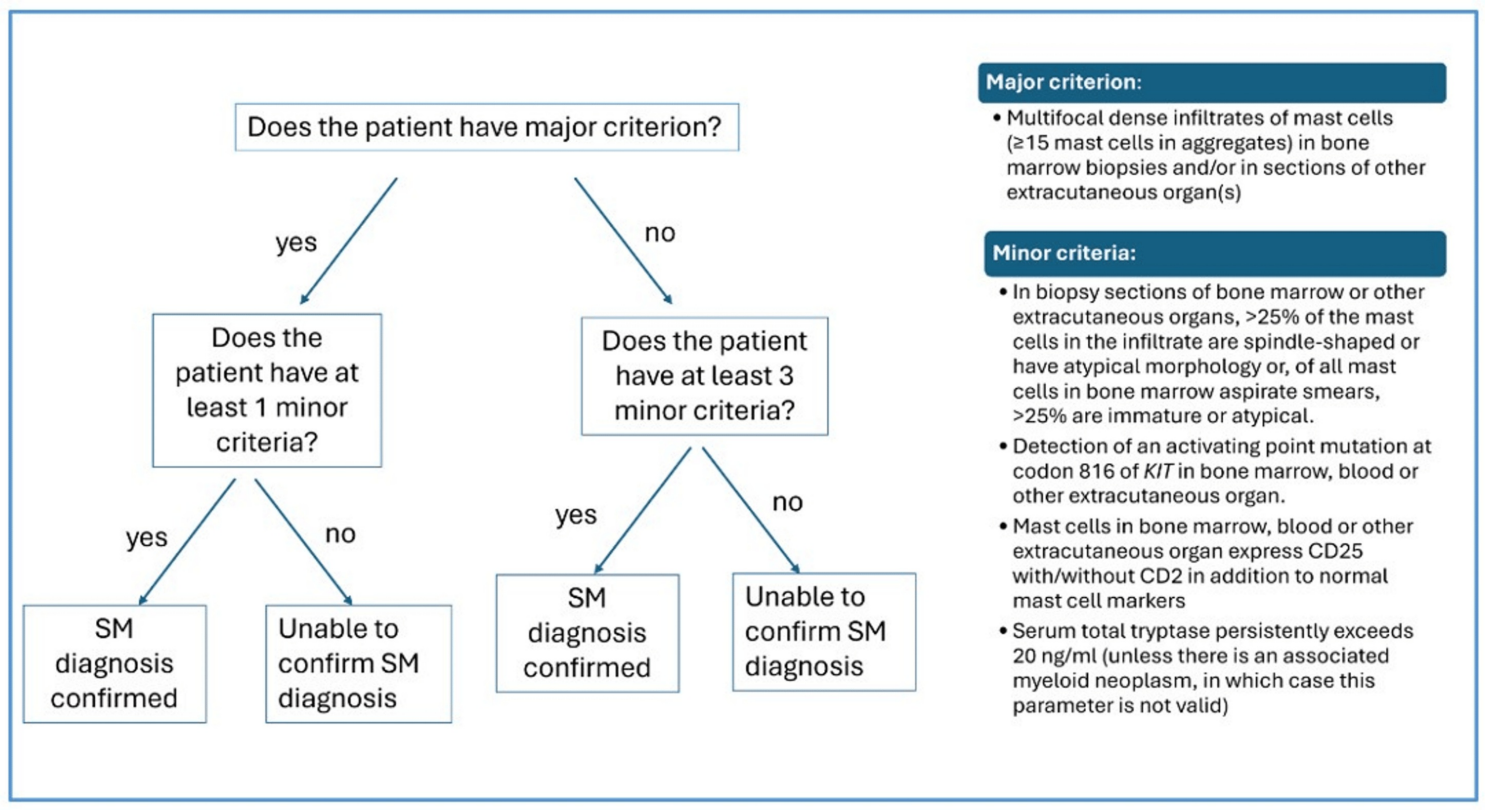
The WHO diagnostic criteria for systemic mastocytosis. Flow chart showing the pathway for diagnosis of systemic mastocytosis according to the WHO diagnostic criteria. SM: systemic mastocytosis This image is created by the authors of this study based on the study by Pardanani [2].

At 38 weeks, she underwent an elective cesarean section under spinal anesthetic with heavy bupivacaine 13 mg and fentanyl 15 mcg. An epidural catheter was inserted but not used. Standard Association of Anesthetists of Great Britain and Ireland (AAGBI) monitoring was used with no invasive lines. The antibiotic choice was intravenous gentamicin 160 mg and clindamycin 900 mg on the advice of the allergy MDT. A 5 mg oxytocin bolus and subsequent oxytocin infusion at 10 units per hour for four hours were used as uterotonics. There was a short episode of cutaneous flushing associated with surgical bowel handling, with no other associated signs or symptoms. She subsequently had a 24-hour stay on the high dependency unit for monitoring and was moved to the labor ward for a further 24 hours. She was discharged with a plan for a bone marrow biopsy to confirm the mastocytosis diagnosis according to the WHO criteria (Figure 1).

## Discussion

There is minimal evidence available for the optimal anesthetic management of pregnant women with SM. A handful of case reports exist in the literature describing labor analgesia, emergency cesarean section under general and spinal anesthesia, and elective cesarean section with a combined spinal epidural [5-8]. These weigh up the risks and benefits of various interventions, with outcomes for the woman and fetus being variable, including significant maternal hypotension and fetal demise. A review article further demonstrates the paucity of evidence for mastocytosis in pregnancy, with only a few studies, all with low numbers of cases [9]. Guidance does exist on the general anesthetic management of patients with SM, with pertinent issues to consider [10]. The authors state that it is impossible to provide a single anesthetic plan due to the multitude of variables, including patient, surgical, and anesthetic factors. Given the relatively narrow spectrum of anesthesia management for elective cesarean section, we have proposed a theoretically optimal plan with a rationale for our decisions described (Table 1). The aim throughout was to decrease potential precipitants and to maintain vigilance and awareness of potential pitfalls.

**Table 1:** Proposed optimal anesthetic plan for elective cesarean section in patients with systemic mastocytosis. TCI: target controlled infusion; HDU: high dependency unit; CVC: central venous catheter

Variables	Plan A - neuraxial anesthesia	Plan B - general anesthesia
Pre-operative	Ensure senior anesthetist and obstetrician.	Ensure senior anesthetist and obstetrician.
	Ensure all potential triggers are removed from theatre environment, e.g., chlorhexidine and latex.	Ensure all potential triggers are removed from theatre environment, e.g., chlorhexidine and latex.
	High-dose antihistamine two weeks prior to operative date.	High-dose antihistamine two weeks prior to operative date.
	Reduce theatre stress, e.g., low lights, music, minimize staff, and provide reassurance to the patient.	Reduce theatre stress, e.g., low lights, music, minimize staff, and provide reassurance to the patient.
	Large-bore cannula - avoid trigger-containing solutions for skin preparation, e.g., chlorhexidine.	Large-bore cannula - avoid trigger-containing solutions for skin preparation, e.g., chlorhexidine.
	Ensure emergency drugs are available, including adrenaline anaphylaxis dose.	Ensure emergency drugs are available, including adrenaline anaphylaxis dose.
Induction	Combined spinal epidural technique.	Ensure airway kit is free of triggers, e.g., tube lubrication is chlorhexidine-free.
	Preparation skin with non-trigger-containing solution, e.g., iodine.	Opiate: alfentanil or fentanyl.
	Spinal: 0.5% heavy bupivacaine and fentanyl.	Induction agent: propofol.
	Epidural top-up: bupivacaine.	Neuromuscular blocker: rocuronium.
	Test block height with cold stick.	Vasopressor: phenylephrine infusion.
	Vasopressor: phenylephrine infusion.	-
Intra-operative	Analgesia: alfentanil or fentanyl.	Analgesia: fentanyl.
	Uterotonics: oxytocin first line as slow IV bolus.	Uterotonics: oxytocin first line as slow IV bolus.
	Antibiotics are to be given after delivery and once hemostasis is achieved.	Antibiotics are to be given after delivery and once hemostasis is achieved.
	-	Maintenance: sevoflurane or propofol/remifentanil TCI.
Emergence	N/A	Reversal: avoid if possible.
Post-operative	Analgesia: oxycodone IV if required.	Analgesia: oxycodone IV if required.
	Use oral route where available.	Use oral route where available.
	Period of observation in a higher-level unit, e.g., HDU, preferably in a side room.	Period of observation in a higher-level unit, e.g., HDU, preferably in a side room.
Other considerations	Consider requirement for blood products.	Consider requirement for blood products.
	Consider the use of a cell saver to reduce the need for blood products.	Consider the use of a cell saver to reduce the need for blood products.
	If central access is required, e.g., for anaphylaxis, ensure the use of CVC without chlorhexidine coating.	If central access is required, e.g., for anaphylaxis, ensure the use of CVC without chlorhexidine coating.

Pre-operative management

Prior to the patient arriving in the hospital, they should be started on oral antihistamines to attempt to decrease the chance of mast cell degranulation. Guidelines suggest that there is no clear time span for this, and trials have not suggested optimal timing [10]. Given the unpredictability of labor and the aim to perform elective cesarean section as close to term as safely possible, it is prudent to consider antihistamines for days to weeks prior to admission.

On the day of surgery, the medical team should consist of a senior anesthetist and obstetrician. This is to manage risk and decrease potential exposure to stressors, as well as decrease the potential for complications, which may in turn precipitate the requirement for potential triggers. The theatre environment should be considered, with the removal of all potential allergen-containing products. Consideration should be given to decreasing emotional and psychological stress, for example, with dimmed lighting or music.

Finally, before the initiation of anesthesia, large-bore IV access should be secured. All preparation material should be checked for triggers. If ultrasound scanning is required, there should be vigilance for the presence of chlorhexidine in lubricant preparations. It is also advisable to prepare diluted adrenaline for ease of use in case of rapid cardiovascular instability.

Induction of anesthesia

Neuraxial anesthesia is the optimum method of anesthesia, requiring fewer drugs both intra-operatively and post-operatively, as well as avoidance of some of the more common anaphylaxis-inducing agents [11]. The lasting analgesic effects of spinal anesthesia can also be beneficial in order to decrease emotional and physical stress. However, some may find neuraxial anesthesia distressing given the requirement to remain awake, and so a balance must be struck with distress mitigated by patient education and psychological support. If general anesthesia is required, a clear plan must be considered at the beginning of all cases.

Reducing exposure to potential triggers is key. Morphine should be avoided due to the comparatively high amounts of histamine released on administration in comparison to fentanyl or oxycodone [12]. Similarly, the use of neuromuscular blocking agents will focus on the reduction of allergenic and histamine-releasing properties [11,13]. Given the requirement for rapid sequence induction, rocuronium is felt to be the safest option.

Amide local anesthetics are already used as standard in obstetric anesthesia, with heavy bupivacaine being an optimum choice for the length of surgery and baricity. Prilocaine would potentially be too short-acting and increase the risk of conversion to general anesthesia [14]. A cold stick for testing block height would allow for minimization of exposure to a potential trigger by ethyl chloride. Care should be taken to clean the stick with non-chlorhexidine-containing solutions. The anesthetist should also be mindful that changes in temperature can also be triggers to mast cell degranulation.

A combined spinal epidural approach would decrease the risk of general anesthesia should the operative time be prolonged or the spinal block be inadequate. Bupivacaine may be used as an epidural top-up to minimise polypharmacy and avoid tachycardia from co-administered adrenaline.

Intra-operative management

All of the principles discussed above apply, and avoidance of triggers is key. In the awake patient, fentanyl or alfentanil can be used for supplemental analgesia. Oxycodone is an optimum choice for the patient under general anesthesia (GA) due to its longer-lasting analgesic effects. Maintenance of anesthesia can be with total intravenous anesthesia (TIVA) or inhalational anesthesia.

At delivery, oxytocin as a slow IV bolus could be used as a first-line uterotonic. It avoids the potential bronchospastic effects of prostaglandin-containing agents and tachycardia associated with ergometrine. Carbetocin could be considered as an alternative; however, it has longer-lasting effects, and so once given, it cannot be stopped should it cause a reaction.

Antibiotics are the main cause of peri-operative anaphylaxis in the United Kingdom, with cefuroxime and gentamicin being the third and fourth most common precipitants [10]. Given the variability in antibiotic prophylaxis and the likelihood that these patients will have a known allergy to antibiotics, a personalized approach is required in conjunction with microbiology colleagues, with avoidance of known allergens. Antibiotic administration should follow delivery, and once surgical hemostasis has been achieved, to prevent any reactions that complicate these periods of known risk.

Emergence and post-operative management

Reversal agents may increase risk. Sugammadex has been shown to be a significant cause of peri-operative anaphylaxis, and neostigmine/glycopyrrolate can have adverse effects on heart rate, as well as anticholinergic effects, which may trigger histamine release [11]. Therefore, it may be prudent to allow time for rocuronium to be metabolized and excreted with quantitative electromyograph monitoring to monitor for adequate return of motor end plate function.

Post-operative analgesia and antiemesis should be via the oral route as much as possible, with IV boluses of oxycodone if required. If GA has been performed, consider local infiltration or TAP blocks by the surgical team. Avoidance of NSAIDs is recommended due to their potential effects on prostaglandin formation and the shift towards production of leukotrienes, inducing bronchospasm. The patient should be monitored in a high dependency setting equipped to deal with sudden cardiovascular collapse secondary to mast cell degranulation and stepped down to a general ward area once suitable.

Other considerations

In addition to histamine, mast cell degranulation can lead to the release of heparin [11]. This can lead to profound coagulopathy, and so consideration should be made for having typed blood products immediately available. Administration of these products can pose a risk, so dynamic decision-making is required. Cell salvage may decrease this risk, allowing for autologous blood return.

If an acute reaction does occur, all standard emergency drugs should be available, and management of any anaphylaxis should be as per advanced life support (ALS) guidelines. This may necessitate central access, and a central venous catheter without chlorhexidine impregnation should be immediately available.

## Conclusions

Systemic mastocytosis is a rare hematological disorder that can present with a wide range of symptoms, including life-threatening anaphylactoid reactions. Reduction of risk through optimization of both the operative environment and anesthetic technique is key to anesthetic management. Vigilance should be maintained throughout the peri-operative period to monitor for signs of acute histamine release and mast cell degranulation. This may require input from colleagues outside of maternity services, including allergy, hematology, and critical care services, to ensure safe delivery of the infant.

The principles described in this elective case can be adapted and applied to the emergency or labor setting. However, there is no single anesthetic that is optimum for pregnant patients with SM, and each patient should have an individualized plan for anesthetic management following consultation with the multi-disciplinary team.
